# Influence of Evoked Compound Action Potential on Speech Perception in Cochlear Implant Users

**DOI:** 10.1016/S1808-8694(15)30095-1

**Published:** 2015-10-19

**Authors:** Mariana Cardoso Guedes, Raimar Weber, Maria Valéria S Goffi Gomez, Rubens Vuono de Brito Neto, Cristina Gomes O Peralta, Ricardo Ferreira Bento

**Affiliations:** a Post-graduate student - Otorhinolaryngology - USP Medical School. Audiologist - Irmandade Santa Casa de Misericórdia de São Paulo. Audiologist - University of São Paulo Medical School, Otorhinolaryngology Department.; b MD. Otorhinolaryngologist. Medical Residency Preceptor - University of São Paulo Medical School, Otorhinolaryngology Department (HCFMUSP).; c PhD in Communication Disorders Sciences - UNIFESP-EPM, Audiologist - University of São Paulo Medical School, Otorhinolaryngology Department.; d Associate Professor of Otorhinolaryngology - Atending Otorhinolaryngologist - University of São Paulo Medical School, Otorhinolaryngology Department.; e M.S. in Audiology. Collaborator - Cochlear Implant Team HCFMUSP.; f Associate Professor of Otorhinolaryngology - University of São Paulo Medical School, Otorhinolaryngology Department. Head of the Ophthalmology and Otorhinolaryngology Department - University of São Paulo Medical School.; Study carried out by the Cochlear Implant Team - Otorhinolaryngology Ward University of São Paulo Medical School, Otorhinolaryngology Department (HCFMUSP).

**Keywords:** cochlear implants, cochlear nerve, speech perception, action potentials, auditory evoked potentials, auditory pathways

## Abstract

Electrically Evoked Compound Action Potential is a measure of synchronous cochlear nerve fibers activity elicited by electrical stimulation of the cochlear implant. The electrophysiological nerve responses may contribute to explain the variability in individual performance of cochlear implant recipients.

**Aim:**

To compare speech perception tests’ performances of cochlear implant users according to the presence or absence of intraoperative neural telemetry responses.

**Material and Method:**

Prospective study design with 100 “Nucleus 24” cochlear implant users divided in two groups according to the presence or absence of intraoperative neural telemetry responses. Speech perception tests were performed after 6 months of continuous use of the device and compared among groups.

**Results:**

Intraoperative action potentials were observed in 72 % of individuals. Open-set sentence test results were better in implant users who had neural telemetry responses when compared to implant users in whom this potential was absent (averages 82.8 % versus 41 %, p = 0.005). There was a strong association between post meningitis-related deafness and absence of intraoperative potentials.

**Conclusion:**

The absence of intraoperative neural telemetry responses was associated with worse performances in speech perception tests and meningitis as etiology of deafness. On the other hand, the presence of these potentials suggests excellent prognosis.

## INTRODUCTION

Individuals with severe to profound sensorineural hearing loss suffer deep damage or reduction in the number of hair cells, and many do not have any benefit from sound amplification through a conventional hearing aid, thus becoming candidates to cochlear implant (CI)^1^.

In this type of device, each electrode directly stimulates the auditory nerve when the electrical current necessary to trigger a hearing sensation passes; and the quantity of electrical current necessary to trigger an auditory sensation is different for each individual and for each stimulation canal. Therefore, the speech processor of each user must be individually adjusted together with each stimulation canal and such process is called “programming” or “mapping”. In adults, power levels must be determined through psychophysical measures (behavioral method). With the progressive increase in energy intensity in each one of the channels, the individual reports on the least intensity at which he/she detects the stimulus (electrical threshold), as well as the maximum intensity allowed without discomfort. In babies, small children or individuals with multiple disorders, such procedure requires techniques that may be inconsistent and non-systematic, because of hearing inexperience or the child’s age. Thus, the use of measures obtained only from the behavioral method to program the speech processor may extend the implant adaptation process because of the difficulty in establishing proper stimulation levels^2^, ^3^, ^4^.

Objective measures used in order to obtain stimulation thresholds and the maximum level of discomfort are being studied and used in an attempt to predict the most adequate stimulation methods for mapping, especially for babies and toddlers^2^, ^3^, ^4^, ^5^, ^6^, ^7^, ^8^.

The Evoked Compound Action Potential - ECAP, reflects the auditory nerve neural activity and may be recorded during surgery, directly from the cochlea by using the implant electrodes as stimulus generator and response recorders, by means of using the specific software^9^. Since then, the correlation between the values obtained objectively and the values researched by the behavioral method (psychoacoustics) are being exhaustively studied, and the threshold obtained from the Neural Response Telemetry - NRT, are used as a routine to program the cochlear implant, especially in children, aiming at predicting the best levels for electrical stimulation^2^, ^6^, ^10^, ^11^, ^12^.

With this method, the speech processor programming became faster and safer, even for babies, toddlers or individuals with multiple disorders since it is not always that conditioned or behavioral responses were consistent and systematic. The programming software allows one to import the threshold levels obtained by NRT and automatically combine them with the psychoacoustic levels obtained through the behavioral test in at least one of the electrodes. Practically all programming methods available and recommended by the manufacturer currently use NRT measures for mapping. They may be adjusted with totally objective methods, or combined with those of correction factor programming^13^, pre-adjusted progressive maps14 or thorough live voice adjustment^12^.

Moreover, the potential testing during surgery is advantageous, since it allows to check the integrity of electrode bundles right after their insertion, and also to investigate neural responses in different cochlear regions. Obtaining data related to the cochlear nerve’s permeability towards electrical stimulation and the way through which some electrical stimulation parameters interact with remaining neural structures still constitutes a challenge. Early determination of exciting neural elements present in the different cochlear pathological processes would also be very useful, since it is supposed that the survival of ganglion cells and other neural elements may constitute one of the causes for variability in relation to speech recognition performance found in implanted individuals^3^, ^15^.

Nonetheless, clinical observation shows that in some patients it is not possible to observe the action potential during surgery, not even in the first months of follow up after activation. In these cases, mapping becomes dependent on the behavioral responses of the individual in order to refer the auditory threshold and the maximum comfort threshold for electrical stimulation in at least three of the 22 electrodes. The more uniform and reliable the answers, the better are the chances of a proper stimulation without discomfort, and this could result in good speech perception.

This study aims at comparing the speech perception performance after 6 months of CI use among patients that have and do not have action potentials found by NRT during surgery, in order to assess the repercussion of response absence in the auditory nerve from the electrical stimulation.

## MATERIALS AND METHODS

The study was approved by the Research Ethics Committee of our institution, under protocol # 633/04.

### Subjects

Between March of 2003 and March of 2005, 102 patients underwent surgery to receive the Nucleus® 24 multichannel cochlear implant. Eighty four patients received models CI24M and CI24K (straight electrodes chain), 7 got the CI24 Contour model (pre-curved electrodes chain for peri-modiolar insertion) and 11 patients that had cochlear ossification proved by tridimensional MRI reconstruction of the cochlear lumen, were implanted with the CI24 Double Array (with two electrodes bundle) model.

Two individuals implanted with model CI24 Double Array were taken off the study later on because they presented inadequate insertion of the electrodes array. Data related to gender, age and time of hearing privation in the patients, as well as etiology, type (pre or post lingual) and modus of hearing loss installation (congenital, sudden or progressive) of the 100 patients studied were collected and are presented in Tables 1 and 2.

**Table 1:** Demographic data related to the hearing of the 100 patients studied.

Males	47 (47,0%)
Age at surgery (years)	20,5 ± 19,7
< 2	3 (3,0%)
2 a 5	33 (33,0%)
6 a 11	17 (17,0%)
12 a 20	8 (8,0%)
≥ 21	39 (39,0%)
Type of hearing loss	
Pre-lingual	62 (62,0%)
Post-lingual	38 (38,0%)
Installation mode	
Congenital	45 (45,0%)
Sudden	34 (34,0%)
Progressive	21 (21,0%)
Hearing loss duration (years)	8,4 ± 8,4
< 5	47 (47,0%)
5 a 9	25 (25,0%)
10 a 14	11 (11,0%)
≥ 15	17 (17,0%)

**Table 2:** Hearing loss etiology of the 100 patients studied.

Unknown	39 (39,0%)
Meningitis	25 (25,0%)
Infectious^1^	7 (7,0%)
Genetic	5 (5,0%)
Ototoxicity	5 (5,0%)
Traumatic	4 (4,0%)
Usher’s syndrome	3 (3,0%)
Otosclerosis	3 (3,0%)
Perinatal anoxia	3 (3,0%)
Widened vestibular aqueduct	2 (2,0%)
Inner ear malformations^2^	2 (2,0%)
Waardemburg syndrome	1 (1,0%)
Ménière’s disease	1 (1,0%)

1: Gestational rubella and toxoplasmosis, Cytomegalovirus and Mumps.

2: Mondini and cochlear hypoplasia.

### Action Potential Study

ECAP investigation was carried out in all individuals during surgery, right after inserting the electrode bundle in the cochlea. We used the NRT 3.1 (Cochlear Co - Denver, CO) software in a microcomputer coupled to the portable programming interface and a SPrint® model speech processor. Recording technique and the subtraction method used to separate the artifact were described by Dillier et al.^9^.

Interpulse interval was fixed in 500µs. Stimulation speed was of 80 Hz with series of 25µs of pulse width. The number of presentations varied between 100 and 200 pulses per second for amplifier gains in 60 and 40db, respectively. Recording window varied from 50 to 150µs according to the optimization of each electrode. The current level of the masking noise was fixed in 10 units above the stimulation level.

The action potential was initially studied in electrodes 20 (apical), 15, 10 (medial), 5 e 3 (basal) as active electrodes, we used distant electrodes + 2 or + 3 as a reference. In case the action potential could not be observed in any of these electrodes, other adjacent pairs were used as active and reference electrodes for the optimization series, until some positive response could be observed. We considered it absence of intraoperative NRT when we did not observe ECAP in any of the tested electrode pairs, considering as bases the characteristics described by Abbas et al.^15^. The maximum intensity used in the test was of 230 current levels.

### Speech Processor Programming

On the day we activate the CI, usually 30 days after surgery, all children who presented NRT responses were mapped according to the action potential thresholds obtained through the linear regression of the amplitude growth curve, carried out by the NRT software (extrapolated threshold) in at least 3 electrodes, Stimulation levels were also stipulated by the programming software, automatically, according to the pre-adjusted progressive maps technique^14^. Afterwards, the processor was turned on and the parameters (minimum and maximum current levels) were adjusted at live voice, in such a way that environmental noise was perceivable and comfortable, without causing pain or discomfort, through the behavioral observation of the patient’s response (combined method).

In order to program the implant of those children that did not have NRT responses, we used behavioral observation in order to obtain the electric threshold. The audiologist was progressively increasing electrical stimulation levels until the child showed some response (sound attention, seeking the sound source). In the case of older children, the threshold was obtained through conditioning by fitting parts. Afterwards, power levels were progressively increased until detecting the intensity at which there were signs of discomfort or adverse reaction from the child in relation to the sound stimulus. Thus, the maximum comfort level was stipulated to be 5 current units below this value. Such procedures were carried out in 4 or 5 channels; the remaining were calculated through the interpolation technique^16^ and then the map was globally adjusted at live voice, in a manner similar to the one described above.

In adults, energy levels were always obtained through the psychoacoustic method, in which the individual responds to stimuli, in each channel, following a loudness scale. By the same token, after initial mapping, the parameters were globally adjusted at live voice.

The remaining stimulation parameters were fixed and identical in both groups: ACE® speech coding strategy at 900 pulses per second per channel and 8 maximum, with pulse width of 25µs. In all children, we activated the ADRO® signal processing to enhance the signal to noise ratio, since they all used the SPrint®^17^model speech processor. In order to program the speech processor we used the R126 v2.1 (Cochlear Co.) processor.

### Outcomes

Tests to assess speech perception were carried out after six months of continuous cochlear implant use (at least eight hours per day) and without orofacial reading or support from signs and gestures. The tests are part of the pre and post-operative assessment protocol and the details of each procedure application were described by Gomez et al.^18^. In this stage we had 83 patients participating, 40 adults and 43 children.

In order to assess speech perception of the children with pre-lingual hearing impairment, we employed the TACAM test - Hearing Capacity Assessment Test, and the adaptation of the GASP - Glendonald Auditory Screening Procedure test, according to each patient’s age^19^, ^20^, ^21^. Results were classified according to the 7 categories of speech perception described by Geers^22^ - Table 3. All post-lingual adults were assessed by the phrases perception test in an open format. We used the list of phrases proposed by Costa^23^ in which the results are expressed in percentages. This test was chosen because it determines the indication of a cochlear implant (≤ 40% during assessment with conventional auditory prosthesis).

**Table 3:** Speech recognition categories in children, according to Geers^22^.

0	Unable to detect speech.
1	Speech detection, however without differentiating stimuli in their supra-segmental aspects.
2	Perception standard (able to differentiate words by their supra-segmental traces).
3	Starting words identification. This child differentiates words, in a closed set, based on phonetic information (words that are identical in duration, but contain multiple spectral differences).
4	Word identification by means of vowel recognition. This child differentiates words in a closed set that differ mainly in vowel sound.
5	Word identification by means of consonant recognition (hand, bread, dog, floor).
6	Word recognition in an open set. This child is able to hear words out of the context and extract much phonemic information, and recognize the word exclusively by means of hearing.

### Statistical Analysis

The mean values of speech perception tests in adults and children were compared among the groups that presented and did not present responses during intra-operative telemetry using the non-parametric Mann-Whitney U test. P values below 0.05 were considered statistically significant.

Data were analyzed with the Statistical Package for Social Sciences (SPSS® for Windows 10.0, SPSS Inc., Chicago, IL) software.

## RESULTS

Intra-operative NRT was observed in 72 (72%) patients.

Auditory Results in Adults: The results from the 40 adults who completed 6 months of CI use are presented on Table 4. Thirteen patients (32.5%) did not show responses at the intra-operative NRT, and had average performance in the phrases recognition test much below the performance of the individuals who responded to the NRT (p = 0.005). Nonetheless, as shown on Table 5, we found a positive association between hearing loss etiology by meningitis and NRT absence during surgery (p = 0.02), and this way we carried out a stratified analysis by etiology (meningitis vs. other causes) according to what is presented on Table 6. Implanted patients with post-meningitis hearing loss had bad results in the open phrases recognition test (mean value of 26.7%, minimum of 0% and maximum of 70%), regardless of the presence or not of intra-operative NRT (p = 0.93). And for those implanted patients that were hearing impaired because of other causes and had NRT present, they had speech perception tests above 90%, while the others presented mean values of 67% and great individual variability (p = 0.02).

**Table 4:** Results of the speech perception tests (phrases in an open context) from the 40 adults that completed 6 months of cochlear implant use.

		NRT	
	Absent (n = 13)	Present (n = 27)	P
Phrases perception in an open context (%)	45,4 ± 34,8	79,3 ± 35,7	0,005

Data presented in average percentage ± standard deviation.

**Table 5:** Prevalence of meningitis-related hearing impairment, according to the presence or not of intraoperative NRT in the 40 adults that completed the 6 months of cochlear implant use.

		NRT	
	Absent (n = 13)	Present (n = 27)	P
Meningitis (n = 12)	7 (53,8%)	5 (18,5%)	0,02
Other causes (n = 28)	6 (46,2%)	22 (81,5%)

**Table 6:** Average correct answers in the open format phrases recognition test in adults with and without meningitis after 6 months of CI use, according to the presence of absence of NRT during surgery.

	Intraoperative NRT		
	Absent	Present	P
Meningitis (n = 12)	27,1 ± 21,4 (n = 7)	26,0 ± 35,8 (n = 5)	0,93
Other causes (n = 28)	66,7 ± 36,7 (n = 6)	91,4 ± 2,5 (n = 22)	0,02

Hearing results in Children: We did not find statistically significant differences in speech performance results among children who had and those who did not have intra-operative NRT -Table 7.

**Table 7:** Results from speech perception in children implanted with or without NRT during surgery.

	Intraoperative NRT		
	Absent (n = 11)	Present (n = 32)	P
Perception category	1,7 ± 1,6	2,3 ± 1,2	0,19
0	2 (18,2%)	3 (9,4%)	
1	4 (36,4%)	5 (15,6%)	
2	3 (27,3%)	9 (28,1%)	
3	0 (0,0%)	9 (28,1%)	
4	1 (9,1%)	6 (18,8%)	
5	1 (9,1%)	0 (0,0%)	
6	0 (0,0%)	0 (0,0%)	

## DISCUSSION

Many authors have analyzed ECAP characteristics and its relation with the current psychoacoustics levels used in electrode mapping. Nonetheless, they failed to find relations between thresholds and potential characteristics and the auditory results from speech perception tests in implanted individuals^3^, ^24^, ^25^.

The hypothesis that ECAP measures could provide some indication as to hearing performance was confirmed in this study, despite we having seen that meningitis was an important factor associated with the absence of potential and the bad results.

No potential found, when isolated from other factors, showed a significant relation with the worst performances in adult speech perception tests, and the absolute speech recognition average was much higher in the group that showed intra-operative NRT responses (all above 90%). Results close to 100% provide excellent speech perception capacity and a good chance of the patient being able to talk on the phone, thanks to the possibility of auditory recognition without the use of orofacial reading or the need for prior knowledge of the context. Individuals that did not respond to the intra-operative NRT test had the most difficulties to understand the day-to-day speech, however they showed an important gain in relation to pre-operative perception (with current mean value of 66% of phrases recognition in an open format).

ECAP represents the synchronic activity of a certain group of neurons and the response amplitude must be proportional to the number of neurons activated by the stimulus. Consequently, the very presence of ECAP may be able to reveal some association with post-operative performance^26^. The survival rate of ganglionary cells and other neural elements may also be considered an explanation for this performance variability in speech recognition that we found^3^. Thus, we may infer that the absence of EACP in cochlear implant users shows this low neural support for stimulus conduction brought about by the device, and this could affect the acoustic information processing necessary for a good speech recognition.

As to the worst performance in open format phrases tests, shown by the group of students in the sample with post-meningitis hearing loss, the findings coincide with those from Blamey et al.^27^, who noticed below the average results in adults with cochlear implant and who had post-lingual hearing loss caused by meningitis. It was suggested that this group would have a lower population of spiral ganglion cells because of lesion characteristics in the auditory system that happen after meningitis, as was also shown in histology studies^28^, ^29^. Lehnhardt and Aschendorff30 calculated that individuals with post-meningitis hearing loss had half the chance to recognize speech without the support from orofacial reading.

It is important to highlight that most individuals with meningitis-related hearing loss presented results in the phrases test below 40%, that is, still below CI indication criterion. In our series, individuals with post-meningitis hearing loss had worse results, even when the potential found by NRT was present. In these patients, the ECAP was not enough to guarantee, by itself, a good performance in speech perception tests. Thus, the triggering of ganglionary cells does not necessarily mean that there will be stimulus recognition in more central levels, because NRT potential recording does not assure that the cognitive mechanisms involved in auditory perception be activated3. For a better assessment of the aspects that may be involved in post-implant and auditory information processing performance, it is necessary, for example, to look for late potentials, such as P300.

In children, we did not observe performance differences between the NRT present and NRT absent groups during surgery. Such fact may be related to the short hearing experience span (6 months), since they all had pre-lingual hearing loss. Moreover, performance in these cases is directly dependent on the therapeutic rehabilitation method employed, as well as its effectiveness. The children implanted in our service come from different Brazilian States, many of them under rehabilitation therapy with local professionals. Although there is a guideline as to the stimulation method and the effective number of therapy sessions, in practice this is heterogeneous, making up an important confusion factor to be considered during the results analysis. Nonetheless, it is possible that after a long term follow up there may be differences in results between the two groups of children.

We did not find data in the literature relating hearing performance in children and the results from the neural telemetry tests. Bevilacqua et al.^31^ and El-Kashlan et al.32 did not find significant differences between hearing performance with cochlear implant in children with meningitis-related hearing loss and children with hearing loss of other etiologies. Nonetheless, both reported a longer delay in speech development in children who had meningitis^26^, ^31^, ^32^.

As far as mapping is concerned, we noticed that the children who did not respond to the NRT and that, therefore, could not be mapped by means of objective or combined techniques require a greater number of returns and a longer time during program. However, since speech perception test results were similar in both groups, we can suggest that the psychoacoustic thresholds study is also valid for babies and small children. It is important to highlight that for the technique to be successful, it is fundamental for the examiners to have experience in childhood audiology and in behavioral observation techniques.

## CONCLUSIONS

- ECAP seen at NRT in adult patients who did not have meningitis, was associated with better results in speech perception tests after 6 months of continuous use of CI, suggesting an excellent short term prognosis.
- Hearing impaired patients with meningitis were associated with higher indices of lack of intraoperative NRT response, and also the worst performances of adult individuals in speech perception tests.
- In children there were no significant differences in speech perception tests between the groups. The marked heterogeneity among the therapies is a confounding factor to be considered, and it is possible that with more time and CI use we may see differences in results.

## References

[1] Bento RF, Brito Neto RV, Castilho AM, Gomez MVSG, Giorgi SB, Guedes MC (2004). Resultados auditivos com o implante coclear multicanal em pacientes submetidos a cirurgia no Hospital das Clínicas da Faculdade de Medicina da Universidade de São Paulo. Rev Bras Otorrinolaringol.

[2] Gordon KA, Gilden JE, Ebinger KA, Shapiro WH (2002). Neural Response Telemetry in 12- to 24-Month-Old Children. Ann Otol Rhinol Laryngol Suppl.

[3] Ferrari DV (2003). Tese (Doutorado em Psicologia) - Instituto de Psicologia. Neurociências e Comportamento.

[4] Thai-Van H, Truy E, Charasse B, Boutitie F, Chanal JM, Cochard N (2004). Modeling the relationship between psychophysical perception and electrically evoked compound action potential threshold in young cochlear implant recipients: clinical implications for implant fitting. Clinical Neurophysiology.

[5] Guedes MC (2003). Medidas de Telemetria de Resposta Neural em Usuários de Implante Coclear Multicanal. Arq Otorrinolaringol.

[6] Gordon K, Papsin BC, Harrison RV (2004). Programming cochlear implant stimulation levels in infants and children with a combination of objective measures. Int J Audiol.

[7] Cafarelli-Dees D (2005). Normative findings of electrically evoked action potential measurements using the neural response telemetry of the Nucleus CI24M cochlear implant system. Audiol Neurootol.

[8] Guedes MC (2005). Telemetria de resposta neural intra-operatória em usuários de implante coclear. Rev Bras Otorrinolaringol.

[9] Dillier N, Lai WK, Almqvist B, Frohne C, Müller-Deile J, Stecker M, Von Wallenberg E. (2002). Measurement of the electrically evoked compound action potential via a neural response telemetry system. Ann Otol Rhinol Laryngol (Suppl 189).

[10] Hughes ML, Brown CJ, Abbas PJ, Gantz BJ (2000). Using electrically evoked compound action potential thresholds to facilitate creating MAPs for children with the Nucleus CI 24M. Adv Otorhinolaryngol.

[11] Seyle K, Brown CJ (2002). Speech Perception Using Maps Based on Neural Response Telemetry Measures. Ear Hear.

[12] Smoorenburg GF, Willeboer C, Van Dijk JE (2002). Speech Perception in Nucleus CI24M Cochlear Implant Users with Processor Settings Based on Electrically Evoked Compound Action Potential Thresholds. Audiol Neurootol.

[13] Bown CJ, Hughes ML, Luk B, Abbas PJ, Wolaver AA, Gervais JP (2000). The relationship between EAP and EABR thresholds and levels used to program the Nucleus CI24M speech processor: Data from adults. Ear Hear.

[14] Novy S (2002 May / June). Nucleus Report.

[15] Abbas PJ, Brown CJ, Shallop JK, Firszt JB, Hughes ML, Hong SH, Stallen SJ (1999). Summary of Results Using the Nucleus CI24M Implant to Record the Electrically Evoked Compound Action Potential. Ear Hear.

[16] Plant K, Law M, Whitford L, Knight M, Tari S, Leigh J (2005). Evaluation of streamlined programming procedures for Nucleus cochlear implant with the contour electrode array. Ear Hear.

[17] James CJ, Blamey PJ, Martin L, Swansom B, Just Y, Macfarlane D (2002). Adaptative dynamic range optimization for cochlear implants: preliminary study. Ear Hear.

[18] Gomez MVSG, Guedes MC, Sant’Anna SBG, Peralta CGO, Tsuji RK, Castilho AM (2004). Critérios de seleção e avaliação médica e audiológica dos candidates ao implante coclear: Protocolo HCFMUSP. Arq Otorrinolaringol.

[19] Bevilacqua MC, Tech EA, Marchesan I.Q, Zorzi J.L, Gomes I.C.D. (1996). procedimento de avaliação de percepção de fala em.

[20] Orlandi ACL, Bevilacqua MC (1998). Deficiência auditiva profunda nos primeiros anos de vida: procedimento para a avaliação da percepção de fala. Pró-Fono.

[21] Lopes AC, Castiquini EAT, Delgado EMC, Bevilacqua MC (2000). Procedimentos de avaliação da percepção de fala em deficientes auditivos. Rev Soc Bras Fonoaudiol.

[22] Geers AE, Geers AE, Moog JS (1994). Volta Review.

[23] Costa MJ, Iorio MCM, Mangabeira-Albernaz PL (2000). Desenvolvimento de um teste para avaliar a habilidade de reconhecer a fala no silêncio e no ruído. Pró-Fono.

[24] Kiefer J, Von Ilberg C, Rupprecht V, Hubner-Egner J, Knecht R (2000). Optimized Speech Understanding with the Continuous Interleaved Sampling Speech Coding Strategy in Patients with Cochlear Implants: effect of variations in stimulation rate and number of channels. Ann Otol Rhinol Laryngol.

[25] Franck KH, Norton SJ (2001). Estimation of Psychophysical Levels Using the Electrically Evoked Compound Action Potential Measured with the Neural Response Telemetry. Capabilities of Cochlear Corporation’s CI24M Device. Ear Hear.

[26] Gantz BJ, Brown CJ, Abbas PJ (1994). Intraoperative Measurements of Electrically Evoked Auditory Nerve Compound Action Potential. Am J Otol.

[27] Blamey P, Arndt P, Bergeron F, Bredberg G, Brimacombe J, Facer G (1996). Factors affecting auditory performance of postlinguistically deaf adults using cochlear implants. Audiol Neurootol.

[28] Nadol JB, Young YS, Glynn RJ (1989). Survival of spiral ganglion cells in profound sensorineural hearing loss: implications for cochlear implantation. Ann Otol Rhinol Laryngol.

[29] Wellman MB, Sommer DD, McKenna J (2003). Sensorineural hearing loss in postmeningitic children. Otol Neurotol.

[31] Bevilacqua MC, Moret ALM, Costa Filho OA, Nascimento LT, Banhara MR (2003). Implantes coleares em crianças portadoras de deficiência auditiva decorrente de meningite. Rev Bras Otorrinolaringol.

[32] El-Kashlan HK, Ashbaugh C, Zwolan T, Telian AS (2003). Cochlear implantation in prelingually deaf children in ossified cochleae. Otol Neurotol.

